# Gantenerumab: an anti-amyloid monoclonal antibody with potential disease-modifying effects in early Alzheimer’s disease

**DOI:** 10.1186/s13195-022-01110-8

**Published:** 2022-11-29

**Authors:** Randall J. Bateman, Jeffrey Cummings, Scott Schobel, Stephen Salloway, Bruno Vellas, Mercè Boada, Sandra E. Black, Kaj Blennow, Paulo Fontoura, Gregory Klein, Sheila Seleri Assunção, Janice Smith, Rachelle S. Doody

**Affiliations:** 1 Department of Neurology, Washington University School of Medicine, St. Louis, MO USA; 2 Dominantly Inherited Alzheimer’s Network (DIAN) and The Knight Family DIAN Trials Unit (DIAN-TU), St. Louis, MO USA; 3 Department of Brain Health, Chambers-Grundy Center for Transformative Neuroscience, School of Integrated Health Sciences, University of Nevada Las Vegas, Las Vegas, NV USA; 4 Product Development, F. Hoffmann-La Roche, Basel, Switzerland; 5 Departments of Psychiatry, Human Behavior, and Neurology, Warren Alpert Medical School of Brown University, Providence, RI USA; 6 Department of Neurology, Butler Hospital, Providence, RI USA; 7 Brown University Center for Alzheimer’s Disease Research, Robert J. and Nancy D. Carney Institute for Brain Science, Providence, RI USA; 8 Department of Geriatric Internal Medicine, UMR 1295 Mixed Unit INSERM – Université Toulouse III Paul Sabatier, Toulouse University Hospital, Toulouse, France; 9 Ace Alzheimer Center Barcelona – Universitat Internacional de Catalunya, Barcelona, Spain; 10 Networking Research Center on Neurodegenerative Diseases (CIBERNED), Instituto de Salud Carlos III, Madrid, Spain; 11 Division of Neurology, Department of Medicine, Sunnybrook Health Sciences Centre, University of Toronto, Toronto, Ontario Canada; 12 LC Campbell Cognitive Neurology Research Unit, Dr Sandra Black Centre for Brain Resilience and Recovery, Hurvitz Brain Sciences Research Program, Sunnybrook Research Institute, University of Toronto, Toronto, Ontario Canada; 13 Department of Psychiatry and Neurochemistry, Institute of Neuroscience and Physiology, Sahlgrenska Academy, University of Gothenburg, Mölndal, Sweden; 14 Clinical Neurochemistry Laboratory, Sahlgrenska University Hospital, Mölndal, Sweden; 15 Roche Pharma Research and Early Development, F. Hoffmann-La Roche, Basel, Switzerland; 16 US Medical Affairs, Genentech Inc., a member of the Roche Group, South San Francisco, CA USA; 17 Product Development, Roche Products Ltd., Welwyn Garden City, UK; 18 Product Development, Genentech Inc., a member of the Roche Group, South San Francisco, CA USA

**Keywords:** Alzheimer’s disease, Amyloid, Biomarkers, Clinical development, Dementia, Gantenerumab, Monoclonal antibody, Neurodegeneration

## Abstract

**Background:**

This review describes the research and development process of gantenerumab, a fully human anti-amyloid monoclonal antibody in development to treat early symptomatic and asymptomatic Alzheimer’s disease (AD). Anti-amyloid monoclonal antibodies can substantially reverse amyloid plaque pathology and may modify the course of the disease by slowing or stopping its clinical progression. Several molecules targeting amyloid have failed in clinical development due to drug-related factors (e.g., treatment-limiting adverse events, low potency, poor brain penetration), study design/methodological issues (e.g., disease stage, lack of AD pathology confirmation), and other factors. The US Food and Drug Administration’s approval of aducanumab, an anti-amyloid monoclonal antibody as the first potential disease-modifying therapy for AD, signaled the value of more than 20 years of drug development, adding to the available therapies the first nominal success since cholinesterase inhibitors and memantine were approved.

**Body:**

Here, we review over 2 decades of gantenerumab development in the context of scientific discoveries in the broader AD field. Key learnings from the field were incorporated into the gantenerumab phase 3 program, including confirmed amyloid positivity as an entry criterion, an enriched clinical trial population to ensure measurable clinical decline, data-driven exposure-response models to inform a safe and efficacious dosing regimen, and the use of several blood-based biomarkers. Subcutaneous formulation for more pragmatic implementation was prioritized as a key feature from the beginning of the gantenerumab development program.

**Conclusion:**

The results from the gantenerumab phase 3 programs are expected by the end of 2022 and will add critical information to the collective knowledge on the search for effective AD treatments.

## Background

Alois Alzheimer was the first to describe amyloid plaques (*miliary foci*) and neurofibrillary tangles in a stained brain section from a person with dementia [1]. Nearly 80 years later, researchers purified insoluble plaques and identified the amino acid sequence of amyloid beta (Aβ) in amyloid plaque cores [2]. These findings enabled the identification of the amyloid precursor protein (*APP*) gene; subsequently, the first pathogenic mutation of the *APP* gene causing familial autosomal-dominant Alzheimer’s disease (AD) was identified [3]. These and other discoveries, including the role of the apolipoprotein E ε4 (*APOE ε4*) gene in increasing the risk of both amyloid aggregation and clinical AD, and the effects of amyloid induction of tau hyperphosphorylation, reduction in cerebral glucose metabolism, and brain atrophy eventually led to the “amyloid hypothesis” of AD proposed by Hardy and Allsop [4] and later updated by Selkoe [5]. This hypothesis holds that processing and deposition of amyloid begin before AD symptoms appear and initiate the underlying pathogenesis of AD. Anti-amyloid therapy development continues to build on the amyloid hypothesis.

Currently, aducanumab is the only anti-amyloid monoclonal antibody approved for the treatment of AD, and approval was based on the US Food and Drug Administration (FDA) accelerated pathway (i.e., aducanumab led to amyloid reduction—a surrogate endpoint reasonably likely to predict clinical benefit) [6]. Several other anti-amyloid monoclonal antibodies are in phase 3 development for AD, including donanemab, lecanemab, and gantenerumab. A phase 3 study with lecanemab was recently reported to have met both its primary endpoint (Clinical Dementia Rating Sum of Boxes [CDR-SB] at 18 months) and all key secondary endpoints with statistical significance [7].

As of September 2022, approximately 2600 participants have been exposed to gantenerumab across several phases of clinical development (i.e., SCarlet RoAD, Marguerite RoAD, DIAN-TU, Open RoAD, GRADUATE I and II, GRADUATION, and POSTGRADUATE trials) [8], with a mean duration of exposure of 2.6 years across these trials. The cumulative patient-years of exposure at the current clinical dose of gantenerumab (equivalent to 1020 mg every 4 weeks) is more than 3100 patient-years.

**Fig. 1:**
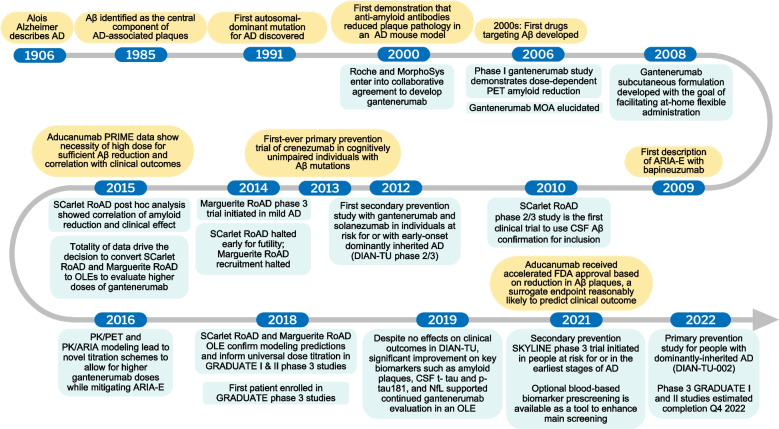
Gantenerumab development and key scientific advancements in AD. Key events and decisions that informed the gantenerumab clinical development program, ultimately leading to the GRADUATE I and II studies—2 ongoing phase 3, global, randomized, parallel-group, placebo-controlled clinical trials evaluating the efficacy and safety of subcutaneous gantenerumab in early AD (i.e., mild cognitive impairment due to AD to mild AD dementia), the launch of secondary prevention trials designed to remove amyloid plaques before symptom onset, and a primary prevention trial designed to prevent formation of amyloid plaques

This review provides an overview of gantenerumab’s clinical development program in the context of the overall history of AD drug development, including clinical trial failures that have informed current drug development efforts and increased researchers’ understanding of AD. This review is structured to parallel the sequence of the drug development process: from the discovery and selection of gantenerumab as a clinical candidate for human testing, through early clinical development, to learnings incorporated from within and outside the program and, finally, to the ongoing phase 3 studies of gantenerumab (GRADUATE I and II) and the implications of this development program for the field (Fig. 1).

## Selection of gantenerumab as a candidate for clinical development

In 2000, F. Hoffmann-La Roche and MorphoSys entered into a collaborative agreement to use MorphoSys’ Human Combinatorial Antibody Library (HuCAL^®^) Fab technology to develop anti-amyloid monoclonal antibodies with potential for AD treatment. Specific anti-amyloid monoclonal antibodies were identified by screening the human phage display library HuCAL^®^-Fab1 [9]. Gantenerumab was selected because of its unique ability to bind to both the N-terminal portions of Aβ and the central amino acids of the Aβ peptide. This characteristic differentiated gantenerumab from other anti-amyloid monoclonal antibodies in development at that time, which all bound to one region of the Aβ sequence. Gantenerumab’s ability to bind both to the flexible N-terminal portions of Aβ and to adjacent central portions of fibrillar Aβ is thought to confer greater binding stability, considered important for therapeutic effect [10].

## Gantenerumab mechanism of action: target engagement of Aβ, especially aggregated forms, and downstream effects

Gantenerumab is a fully human Aβ immunoglobulin G1 antibody designed to promote clearance of amyloid plaques in the brain, through peptide aggregate dissociation and fibrillar Aβ clearance [9, 11]. The human immunoglobulin G1 backbone promotes Fc gamma receptor-mediated microglial phagocytosis of aggregated Aβ [9, 11]. Electron microscopy has shown that gantenerumab binds to Aβ fibrils within Aβ plaques ex vivo, while immunofluorescence staining revealed binding of gantenerumab to Aβ in the brains of patients with AD [9, 12]. Live-cell imaging in postmortem AD brain tissue suggests that removal of fluorescent-labeled gantenerumab bound to Aβ plaques occurs through Fc gamma receptor/microglia-mediated phagocytosis, followed by lysosomal degradation [11].

**Fig. 2:**
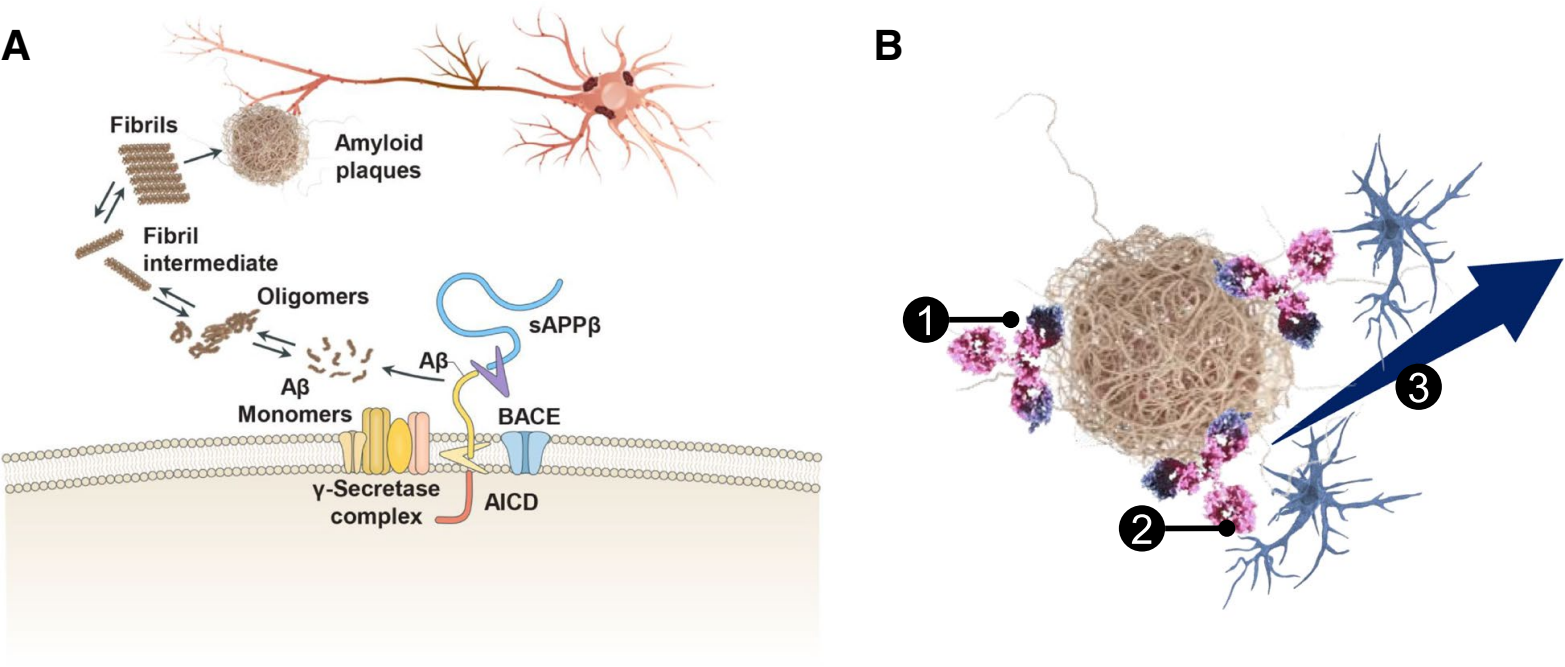
Amyloid aggregation in AD and the mechanism of action of gantenerumab. **A** The amyloid hypothesis of AD involves the accumulation of Aβ-soluble monomers and oligomers that aggregate into insoluble fibrils and amyloid plaques. Aggregated species of Aβ damages neurons and can increase oxidative stress, inflammation, mitochondrial dysfunction, and neuronal loss. **B** Based on the findings from in vitro studies, the mechanism of action of gantenerumab is thought to stem from the clearance of Aβ plaques by antibody-induced cell-mediated phagocytosis, dissociation of Aβ peptide aggregates by direct resolution, and neutralization of neurotoxic Aβ oligomers. Gantenerumab binds to all types of aggregated neurotoxic Aβ species, with the highest affinity to the fibrillar forms and plaques. After binding to aggregated amyloid (1), the Fc gamma receptor on microglia binds to the human immunoglobulin G1 backbone of gantenerumab, engulfing Aβ plaques (2), and, then, phagocytosis by microglia and removal of aggregated Aβ (3). **A** Adapted with permission from Panza F, et al. Nat Rev Neurol. 2019;15(2):73-88

Gantenerumab’s affinity is highest for aggregated forms of Aβ (i.e., fibrils, plaques) and soluble oligomers. In vitro evidence suggests that binding to oligomers occurs, neutralizing oligomer toxicity [9]. The data described above suggest that gantenerumab has 2 important effects on aggregated Aβ—interruption of aggregation growth and activation of microglial phagocytosis. Figure 2 depicts the amyloid hypothesis of AD and gantenerumab’s mechanism of action.

In addition to its impact on Aβ, gantenerumab has effects on multiple biomarkers of AD pathology and neurodegeneration in clinical trials, including dose- and time-dependent reductions in cerebrospinal fluid (CSF) levels of total tau (t-tau), phosphorylated tau (p-tau) reduction, decreases in the synaptic biomarker neurogranin [13, 14], and neurofilament light chain (NfL) reduction [14], which provide evidence supporting gantenerumab’s impact in several biological aspects of AD pathophysiology.

### Amyloid-related imaging abnormalities: a finding related to the effects of anti-amyloid monoclonal antibodies targeting fibrillar Aβ

Anti-amyloid monoclonal antibodies that substantially remove Aβ plaques are associated with an adverse event (AE) known as amyloid-related imaging abnormalities (ARIA) with edema (ARIA-E) or with microhemorrhage or superficial siderosis (ARIA-H) [15, 16]. ARIA was first observed in 2009 in clinical trials with bapineuzumab, a humanized anti-Aβ monoclonal antibody binding to monomeric, oligomeric, and fibrillar forms of Aβ, as well as plaques [17]. ARIA has since been seen with all anti-amyloid monoclonal antibodies that substantially remove Aβ plaques [18] and is an AE considered “on target” because it is related to amyloid removal. It is therefore described here, alongside the mechanism of action of gantenerumab and other amyloid-removing monoclonal antibodies. ARIA is hypothesized to be related to temporarily increased vascular permeability that occurs due to enhanced trafficking of parenchymal Aβ to the perivascular space and/or leakage of blood vessels after vascular Aβ clearance [19].

With maintained antibody-mediated amyloid clearance, the vessels may regain their structural integrity and ARIA incidence typically decreases after 6 to 9 months of treatment with a monoclonal antibody [19, 20]. The risks of ARIA-E and ARIA-H are associated with *APOE ε4* genotype in a dose-dependent fashion; homozygotes show the highest rates of ARIA and heterozygotes exhibit more ARIA than non-carriers [18]. APOE4 leads to blood-brain barrier dysfunction, predicting cognitive decline [21]. Other risk factors for ARIA include advanced age, amyloid burden, microbleeds present at baseline, hypertension, and anticoagulant use [19, 20, 22–24].

## Phase I clinical trials of gantenerumab: demonstrating target engagement and initial ARIA observations that influenced the clinical development program

To date, 543 individuals have participated in phase 1 studies of gantenerumab: 4 bioavailability studies in healthy participants, 3 single ascending dose studies, and 2 multiple ascending dose (MAD) studies. Of these phase 1 study participants, 406 healthy volunteers and 101 patients with AD received gantenerumab [25–28].

One phase 1 randomized, double-blind, placebo-controlled MAD study evaluated the safety, tolerability, and pharmacokinetic (PK) and pharmacodynamic (PD) characteristics of gantenerumab in patients with mild to moderate AD dementia [11].

Additionally, a phase 1 positron emission tomography (PET) substudy of the main MAD study was conducted on 16 participants to assess treatment-related reductions in brain Aβ levels. The mean (95% CI) percent change from baseline relative to placebo in cortical brain amyloid level was − 15.6% (− 42.7 to 11.6%) for the 60 mg group and − 35.7% (− 63.5 to − 7.9%) for the 200 mg group. This study demonstrated a dose-dependent reduction in PET Aβ plaque level after treatment with gantenerumab, laying the groundwork for clinical efficacy studies. Two patients in the 200 mg group developed ARIA-E and ARIA-H in the brain regions with the highest level of Aβ reduction; the ARIA-E events were transient. ARIA was a new phenomenon in the field at the time of this phase 1 MAD study, and its clinical impact was not fully understood. Hence, to ensure patient safety by mitigating ARIA risk, conservative doses were selected for the earliest gantenerumab clinical efficacy studies (i.e., 105 mg) and were uptitrated to a higher dose (i.e., 225 mg) if ARIA did not appear [11]. These doses were later recognized to be approximately 5- to 10-fold below the dose currently thought to be necessary for efficacy (specifically, in clinical trials with a limited duration of up to 2 years).

### Rationale for the development of a subcutaneous formulation of gantenerumab for clinical studies

Most current pharmacological AD treatments are orally administered, and most monoclonal antibodies are administered intravenously (IV). However, subcutaneous (SC) administration may be preferred, as some patients with AD may have less mobility, encounter challenges to obtaining IV access, and benefit from at-home administration by a professional or non-professional care partner. Furthermore, IV infusion increases costs to the healthcare system and imposes an additional strain on the patient and care partner. SC formulation of gantenerumab was therefore prioritized early in development and was implemented before the initiation of the SCarlet RoAD study in 2010.

To examine the tolerability of the SC approach in the volumes necessary to deliver target doses of gantenerumab, a phase 1 randomized, open-label, single-dose (300 mg), placebo-controlled crossover study of healthy volunteers was conducted between 2016 and 2017. On an analog pain scale from 0 to 100, the mean (SD) scores for 5- and 15-s gantenerumab injections were 22.16 (23.09) and 14.96 (18.38), respectively, whereas the mean (SD) scores for 5- and 15-second placebo injections were 26.58 (27.83) and 14.16 (20.62), respectively [29]. The mean score on this scale was comparable between SC gantenerumab and placebo abdominal injections. Pain subsided within 5 min of dosing. AEs were mostly mild injection site reactions (expected with SC administration). Together, the preclinical and early-phase clinical studies of SC gantenerumab supported its potential utility in AD and provided baseline data on initial dosing and administration sufficient to guide subsequent studies.

## SCarlet RoAD and Marguerite RoAD: the first phase 2/3 clinical trials that paved the way for the future development of gantenerumab

The SCarlet RoAD trial [13, 25] was originally designed as a multicenter, randomized, double-blind, placebo-controlled phase 2 study investigating the efficacy and safety of conservative doses of SC gantenerumab (105 mg or 225 mg every 4 weeks) in participants with prodromal AD over a 2-year period [13, 25]. The inclusion criteria for this first study to recruit a purely prodromal AD population [30] included patients with a recent gradual decline in memory function, impaired episodic memory on testing, a CDR scale global score of 0.5, a CDR memory score of 0.5 or 1, and biomarker evidence of AD pathology (CSF Aβ1–42 level < 600 pg/mL). Importantly, this trial was the first to require amyloid biomarker confirmation for study inclusion. Requiring this confirmation enhanced the prevalence of AD amyloid plaques in the study population and affirmed that the population was appropriate for treatment with anti-amyloid therapy [13].

## Clinical trial methodology considerations for Scarlet RoAD phase 2

**Fig. 3:**
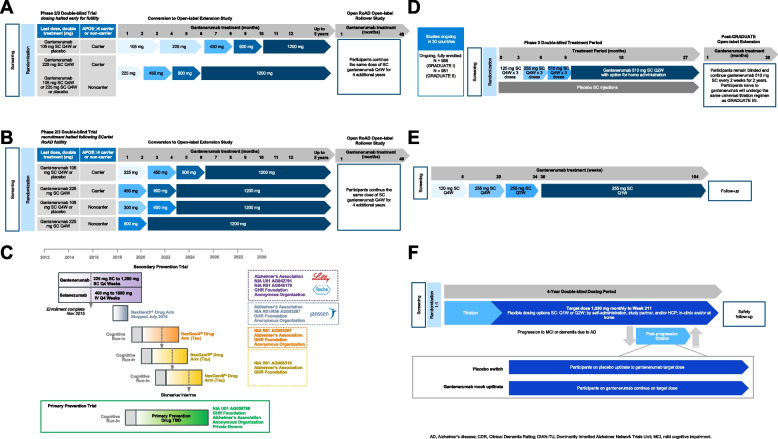
**A** SCarlet RoAD (prodromal AD, CDR 0.5, ClinicalTrials.gov identifier: NCT10224106) study design: conversion from a gantenerumab phase 2/3 study in prodromal AD to an open-label extension and rollover into the Open RoAD open-label study. **B** Marguerite RoAD (mild AD, CDR 1.0, ClinicalTrials.gov identifier: NCT02051608) study design: conversion from a gantenerumab phase 3 study in mild AD to an open-label extension and rollover into the Open RoAD open-label study. **C** The Knight Family DIAN-TU dominantly inherited autosomal Alzheimer’s disease (prevention, prodromal or mild AD; ClinicalTrials.gov identifier: NCT01760005) study design: conversion from a gantenerumab phase 2/3 study to an exploratory open-label extension with dose escalation in asymptomatic or mild AD from DIAN-TU. **D** GRADUATE I and II (ClinicalTrials.gov identifiers: NCT03444870 and NCT03443973) phase 3 studies: two global, parallel, multicenter, randomized, double-blind, placebo-controlled studies of gantenerumab in patients with early AD (prodromal or MCI-AD to mild AD), and the post-GRADUATE (ClinicalTrials.gov identifier: NCT04374253) open-label extension study. **E** GRADUATION (ClinicalTrials.gov identifier: NCT04592341) study design: a multicenter, phase 2, open-label, single-arm, pharmacodynamic study to evaluate once-weekly subcutaneous gantenerumab dosing. After titration, at-home administration by care partners or home nurses is possible, allowing flexibility for patients. **F** SKYLINE (ClinicalTrials.gov identifier: NCT05256134) study design: a phase 3, randomized, double-blind, placebo-controlled secondary prevention trial of gantenerumab in participants at risk or at the earliest stages of AD. **C** Adapted with permission from “As DIAN wraps up anti-aβ drug arms, it sprouts tau, primary prevention arms.” 2019. Available from AlzForum at https://www.alzforum.org/news/conference-coverage/dian-wraps-anti-av-drug-arms-it-sprouts-tau-primary-prevention-arms

The Scarlet RoAD study, which began in 2010 and was stopped for futility in December 2014, utilized learnings from the field in recruiting patients at an early stage on the AD continuum (i.e., prodromal AD) and in requiring confirmed amyloid positivity. The initial doses (Fig. 3A) in this study (105 mg or 225 mg every 4 weeks) were chosen based on clinical data available at the time, which suggested that a dose above 100 mg was necessary for efficacy and a dose below 330 mg would minimize ARIA incidence, especially in *APOE ε4* carriers [13, 31]. Other studies (e.g., phase 3 trials of bapineuzumab [17, 19]) later indicated that ARIA-E was mostly asymptomatic and was more likely to occur in *APOE ε4*-positive individuals and that dosing could continue while these events were monitored. Given the observed relationship between *APOE ε4* genotype and ARIA, patients in the SCarlet RoAD trial were randomized to different doses based on *APOE ε4* genotype: *APOE ε4* homozygotes to 105 mg gantenerumab or placebo and *APOE ε4* heterozygotes and non-carriers to 225 mg gantenerumab or placebo [13].

Given the positive results of the phase 1 gantenerumab PET substudy showing some Aβ removal with supportive safety outcomes, SCarlet RoAD was converted from a phase 2 to a phase 3 trial in 2012 after 799 patients had enrolled [13, 25], and a second phase 3 study in mild AD dementia, Marguerite RoAD, was initiated. Marguerite RoAD was a multicenter, randomized, double-blind, placebo-controlled, parallel-group study evaluating the efficacy and safety of SC gantenerumab (105 mg or 225 mg every 4 weeks) in participants with mild AD dementia over a 2-year period (Fig. 3B) [32].

In late 2014, a preplanned interim futility analysis of the SCarlet RoAD phase 3 trial was conducted after 50% of patients completed at least 2 years of treatment. No differences were observed between the groups on the primary clinical endpoint—change from baseline in the CDR-SB (gantenerumab 105 mg: 0.10-point change [*P* = 0.67]; gantenerumab 225 mg: 0.18-point change [*P* = 0.45]) [13]. Secondary endpoints showed no statistically significant differences from placebo. CSF biomarker results showed a numeric increase in Aβ1–42 and a numeric decrease in t-tau for gantenerumab vs placebo at weeks 52 and 104. There was a statistically significant decrease in p-tau with gantenerumab treatment at week 104 (*P* = 0.01). The reduction in the p-tau/Aβ1–42 ratio was significant for gantenerumab vs placebo at week 52 (*P* = 0.03) and at week 104 (*P* < 0.01; Table 1) [33]. ARIA incidence increased in a dose- and *APOE ε4* allele-dependent manner, although most cases were asymptomatic. Injection site erythema events were mild to moderate and occurred in 29/271 patients (10.7%) in the gantenerumab 105 mg group and in 35/260 patients (13.5%) in the gantenerumab 225 mg group [13].

**Table 1:** Completed trials of gantenerumab

Study name, design, and NCT	Study population	Gantenerumab dose	Primary endpoint	Other key findings
**SCarlet RoAD** **Phase 2/3, global double-blind placebo-controlled study** (study start date: 2010) (NCT01224106)	*N* = 797 Prodromal AD^**a**^, 50–85 years, CSF Aβ1–42 confirmed pathology < 600 pg/mL	Gantenerumab 105 mg SC Q4W Gantenerumab 225 mg SC Q4W Placebo	**Change from baseline in CDR-SB at 2 years in the gantenerumab group:** 105 mg, 1.69 (*P *= .67) 225 mg, 1.73 (*P *= .45) Dosing stopped due to preplanned interim futility in 2014 Study converted to OLE in 2015	**CSF biomarkers in gantenerumab group** (*n* = 209); change from baseline at 104 weeks: **Aβ(1–42):** gantenerumab 105 mg, -1.06% (*P *= .98); gantenerumab 225 mg, 7.55% (*P *= .09) **t-tau:** gantenerumab 105 mg, – 1.08% (*P *= .05); gantenerumab 225 mg, – 2.91% (*P *= .02) **p-tau:** gantenerumab 105 mg, – 5.61% (*P *≤ .001); gantenerumab 225 mg, – 7.15% (*P *≤ 0.001) **neurogranin:** gantenerumab 105 mg, – 4.58% (*P *= .79); gantenerumab 225 mg, – 11.76 (*P *= .18) **PET substudy** [34] (*n* = 115): **No changes in CSF** Aβ1–42 were found for either group **Gantenerumab 105 mg:** – 4.85% change from baseline in CSF p-tau (*P *< .01) at week 104; – 1.45% change from baseline in CSF t-tau (*P *< .01) at week 104; 0.19% change from baseline in composite standardized uptake value on amyloid PET at week 100 **Gantenerumab 225 mg:** – 7.52% change in CSF p-tau (*P *< .01) at week 104; – 2.94% change from baseline in CSF t-tau (*P *< .01) at week 104; – 5.37% change from baseline in composite standardized uptake value on amyloid PET at week 100 **Safety** One adverse event, injection site erythema, had > 5% occurrence and 2X greater than placebo: gantenerumab 105 mg, 10.7%; gantenerumab 225 mg, 13.5%; placebo, 1.1%
**Marguerite RoAD Phase 3 double-blind, placebo-controlled parallel-group study** (study start date: 2014) (NCT02051608)	*N* = 389 50–90 years Probable mild dementia^**a**^ CSF Aβ1–42 confirmed pathology < 700 pg/mL	Gantenerumab SC Q4W uptitrated to 225 mg at week 28 if no confirmed ARIA (Fig. 3) Placebo	**Change from baseline in ADAS-Cog13 scores at week 104 vs placebo** **Change from baseline in ADCS-ADL scores at week 104 vs placebo** Terminated early following SCarlet RoAD futility analysis; study converted to OLE in 2015 Mean time on treatment = 66 weeks	**CSF biomarkers in gantenerumab group** (*n* = 12) median % from baseline at 104 weeks: **Aβ(1–40)**: – 10.03 [– 18.08; – 2.21], *P *= .584 **Aβ(1–42)**: 7.15 [– 8.52; 15.97], *P *= .123 **t-tau**: – 6.17 [– 12.72; 1.25], *P *= .184 **p-tau:** – 16.33 [– 22.84; – 4.49], *P *= .053
**SCarlet RoAD** **Open-label extension study** (study start date: 2015) (NCT01224106)	*N* = 154 Patients enrolled in the SCarlet RoAD study (NCT01224106)	Gantenerumab SC Q4W, uptitrated to 1200 mg based on *APOE ε4* status (Fig. 3)	**Safety** AEs were mostly mild to moderate. 31.2% of patients experienced ISR; one patient discontinued due to ISR, which was reported as mild to moderate.	
**Marguerite RoAD** **Open-label extension study** (study start date: 2015) (NCT02051608)	*N* = 225 Patients enrolled in the Marguerite RoAD study (NCT02051608)	Gantenerumab SC Q4W uptitrated to 1200 mg based on *APOE ε4* carrier status (Fig. 3)	**Safety** AEs were mostly mild to moderate. 29.8% of patients experienced ISR; 10.7% of patients discontinued due to AE.	
**SCarlet RoAD OLE** (NCT01224106) **and Marguerite RoAD OLE** (NCT02051608) **PET substudy: exploratory analyses** (study start date: 2015)	(*n* = 67) Prodromal AD^a^, 50–85 years, CSF Aβ1–42 confirmed pathology **Three cohorts:** 1) Pooled SR gantenerumab 105 mg or 225 mg or placebo Q4W (*n* = 19) 2) MR double-blind placebo (*n* = 27) 3) MR double-blind active gantenerumab 105 mg or 225 mg (*n* = 21)	Gantenerumab 1200 mg SC Q4W (Fig. 3)	**Change from baseline in mean Aβ PET centiloid values** **52 weeks:** pooled SR, – 21; MR double-blind placebo, – 42; MR double-blind active, – 48 **104 weeks:** pooled SR, – 34; MR double-blind placebo, – 71; MR double-blind active, – 62 **36 months:** pooled SR, – 57.0; MR double-blind placebo, – 90.3; MR double-blind active, – 74.9	At 104 weeks, 51% of patients were below the amyloid positivity threshold. At the 36-month follow-up (140 weeks total), 80% of participants were below the amyloid positivity threshold.
**DIAN-TU-001** **Public-private collaboration, randomized, placebo-controlled, multi-arm phase 2/3 trial** (study start date: 2012) (NCT01760005)	*N* = 142 Asymptomatic or mildly symptomatic participants with DIAD	Gantenerumab SC uptitrated to 1200 mg Q4W (see Fig. 3) Solanezumab uptitrated to 1600 mg Placebo	DIAN-MCE evaluated via Bayesian multivariate CPR compared with pooled placebo: **Gantenerumab** probability CPR < 1 = 0.144 (no treatment benefit)	**No benefit with gantenerumab in the following:** MMRM change from baseline in each component of DIAN-MCE MMRM change from baseline in CDR-SB MMRM change from baseline in Functional Assessment Scale **Changes in biological endpoints from baseline to year 4: between-group difference (gantenerumab vs placebo)** **PiB-PET Aβ:** 24.3% (12.7 + 11.6) decrease with gantenerumab(*P *< .001) **CSF total Aβ42:** 42.6% (19.3 + 23.3) increase with gantenerumab (*P *< .001) **CSF total tau:** 20.6% (15.3 + 5.3) decrease with gantenerumab (*P *< .001) **Phospho-tau181:** 32.8% (23.4 + 9.4) decrease with gantenerumab (*P *< .001) **CSF NfL:** 2.2% (3.9 – 1.7) slowed increase with gantenerumab (*P *< .05) **Cortical metabolism** measured with ^18^F-FDG-PET (no difference) **Precuneus thickness and hippocampal volume** (no difference) **Safety** The most common adverse event with gantenerumab was injection-site reactions (90%; *P *< .0001 vs placebo). No other adverse event occurred statistically significantly more with gantenerumab than placebo.

*Abbreviations*: ^*18*^*F-FDG-PET* ^18^F-fluorodexyglucose positron emission tomography, *Aβ* amyloid-beta, *Aβ(1–40)* amyloid beta protein fragment 1-40, *Aβ(1–42)* amyloid beta protein fragment 1-42, *AD* Alzheimer’s disease, *ADAS-Cog13* Alzheimer’s Disease Assessment Scale–Cognitive Subscale 13, *ADCS-ADL* Alzheimer’s Disease Cooperative Study–Activities of Daily Living instrumental subscale, *AE* adverse event, *APOE ε4* apolipoprotein E ε4, *ARIA* amyloid-related imaging abnormalities, *CDR* Clinical Dementia Rating, *CDR-SB* Clinical Dementia Rating Scale–Sum of Boxes, *CPR* cognitive progression ratio, *CSF* cerebrospinal fluid, *DIAD* dominantly inherited Alzheimer’s disease, *DIAN-MCE* Dominantly Inherited Alzheimer Network–Multivariate Cognitive Endpoint, *DIAN-TU* Dominantly Inherited Alzheimer Network–Trials Unit, *ISR* injection site reaction, *mg* milligram, *mL* milliliter, *MMRM* mixed models for repeated measures, *MR* Marguerite RoAD, *NCT* National Clinical Trial number, *NfL* neurofilament light chain, *OLE* open-label extension, *PET* positron emission tomography, *pg* picogram, *PiB-PET* Pittsburgh compound-B positron emission tomography, *p-tau* phosphorylated tau, *Q4W* every 4 weeks, *SC* subcutaneous, *SR* SCarlet RoAD, *t-tau* total tau.

^a^International Working Group Criteria for Prodromal AD [30]

Based on the futility assessment from SCarlet RoAD, dosing was interrupted, but the study was not terminated; participants continued to be followed for safety, biomarkers, and clinical assessments. In addition, although recruitment was stopped for the Marguerite RoAD trial, dosing continued. Despite the futility results, both SCarlet RoAD and Marguerite RoAD yielded new, important information regarding gantenerumab and AD that informed future trial designs.

## SCarlet RoAD and Marguerite RoAD conversions to open-label extension studies: a path forward to apply lessons learned

The prodromal AD population in SCarlet RoAD was notable for a relative lack of progression in the placebo group, making it more challenging to show treatment effects in this progressive disease. Even before SCarlet RoAD was unblinded, an AD progression model had been built for CDR-SB using data from the Alzheimer’s Disease Neuroimaging Initiative to distinguish between “slow” and “fast” progressors [35]. Using that model, SCarlet RoAD data in fast progressors showed a dose-dependent correlation between exposure and amyloid reduction, and exposure-dependent slowing of cognitive decline [13]. Contemporaneously, data from the aducanumab PRIME study that evaluated IV doses of up to 10 mg/kg indicated that dose- and time-dependent Aβ reductions were associated with clinical benefit in AD [36].

**Fig. 4:**
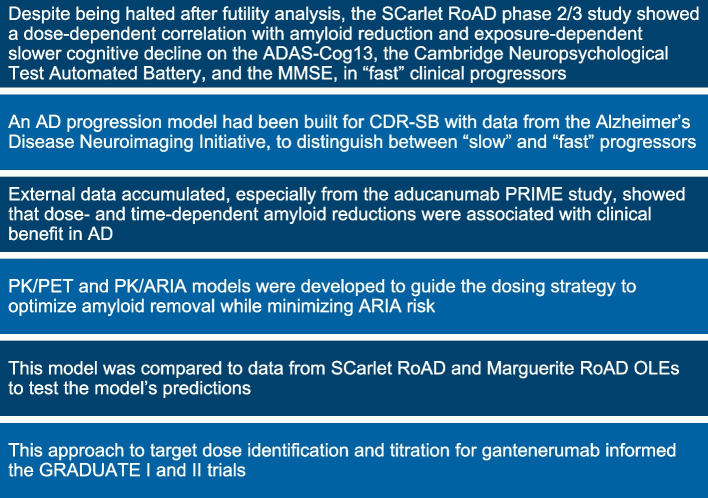
Scientific lessons that informed the updated dosing strategy of gantenerumab

These prespecified and post hoc analyses of the SCarlet RoAD and Marguerite RoAD (Marguerite RoAD data not described here) indicated dose-dependent amyloid reduction and dose-/exposure-dependent clinical signals (Fig. 4). Based on these findings, SCarlet RoAD and Marguerite RoAD were converted into open-label extension (OLE) studies in 2015 to investigate the effects of higher doses of gantenerumab on amyloid reduction and ARIA incidence using various titration regimens to reach a higher target dose. All patients in these studies were offered the opportunity to participate in the OLE, even those who had progressed beyond prodromal or mild dementia.

The approach to target dose identification and titration for the OLE studies was further informed by a PK/PET model developed to maximize plaque removal while minimizing ARIA incidence [37]. Patients in the OLE were escalated to up to a 1200-mg monthly dose based upon this information.

## SCarlet RoAD and Marguerite RoAD OLE PET substudy: biomarker-confirmed, exposure-dependent Aβ plaque removal

The OLE studies included a PET substudy to empirically test and further inform the PK/PET model for the predicted higher dose of gantenerumab: 1200 mg every 4 weeks in patients with prodromal to moderate AD dementia [38, 39]. Data analyses from these substudies are presented based on 3 cohorts: (1) SCarlet RoAD patients, (2) Marguerite RoAD patients on gantenerumab, and (3) Marguerite RoAD patients on placebo.

**Fig. 5:**
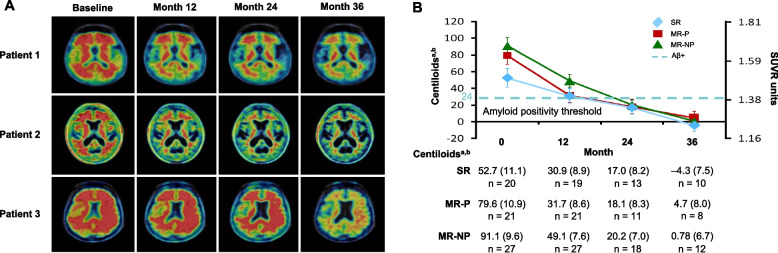
Aβ plaque reduction with gantenerumab in basal ganglia before and up to 36 months after treatment, and reduction of amyloid burden toward zero centiloids after 36 months of open-label therapy. **A** Axial florbetapir brain PET images from 3 patients displaying different rates of reduction of Aβ plaques from OLE baseline to OLE week 52 and OLE week 104. Axial slices are at the level of the basal ganglia. PET images were obtained 50 min post-injection, standardized uptake value data with the cerebellar cortex as the reference region. ^a18^F-Florbetapir amyloid PET. ^b^LS mean (SE) analyzed using a mixed model for repeated measures; 1200 mg subcutaneous gantenerumab was administered every 4 weeks during the OLE studies. **B** Marked and consistent reduction of amyloid load in patients receiving high-dose gantenerumab. Marked reduction of amyloid-β plaques in patients receiving high-dose gantenerumab, and consistent reduction of amyloid-β plaques in all patient groups. **B** adapted from Klein G, et al. J Prev Alzheimers Dis. 2021;8(1):3-6, licensed under CC BY 4.0

An interim analysis of 67 participants (SCarlet RoAD double-blind, active cohort; Marguerite RoAD double-blind, active cohort; and Marguerite RoAD double-blind, placebo cohort) conducted 2 years into the OLE confirmed that higher doses (up to 1200 mg) were associated with greater Aβ plaque reduction (Fig. 5) [39]. At OLE years 1, 2, and 3, 37%, 51%, and 80% of patients, respectively, had Aβ plaque levels below the Aβ positivity threshold (previously established as 24 centiloids, which corresponds to 1.40 standardized uptake value units) [39, 40]. Furthermore, gantenerumab demonstrated continued reduction of Aβ plaques in all 3 cohorts approaching zero centiloids (i.e., the level of amyloid negativity). The mean (SE) centiloid values in cohorts 1, 2, and 3 were − 4.3 (7.5), 0.8 (6.7), and 4.7 (8.0), respectively; this was a change from the baseline of − 57.0 (10.3), − 90.3 (9.0), and − 74.9 (10.5) centiloids, respectively. These results demonstrated that despite the different mean baseline centiloid values in the 3 cohorts, prolonged treatment with 1200 mg gantenerumab administered every 4 weeks continued to reduce Aβ plaque levels below the Aβ positivity threshold, achieving a PD effect several-fold higher in magnitude than the first PET study that used much lower doses [38].

At approximately 2 years of the mean treatment duration, rates of ARIA-E events in the SCarlet RoAD OLE [41] and Marguerite RoAD OLE [42] ranged from 13.6 to 38.3% and may have been influenced by several risk factors, including (but not limited to) previous exposure to gantenerumab in the double-blind phase, rate of uptitration, and *APOE ε4* carrier status [37]. The OLE studies helped demonstrate that a higher target dose had an acceptable tolerability profile irrespective of *APOE ε4* genotype.

In patients treated with gantenerumab in the SCarlet RoAD and Marguerite RoAD OLEs, 65% of ARIA-E cases were clinically asymptomatic. Most reported symptoms were non-specific (e.g., headache, dizziness, confusion). Eight participants (2.1%) had serious symptoms, which included seizure/epilepsy, confusion, hemiplegia, cerebral hematoma, and ischemic stroke. In all cases, complete symptom resolution was observed upon dose interruption [8, 18].

## Dominantly Inherited Alzheimer Network Trials Unit

In 2011, as AD studies in patients who had non-familial cases of early sporadic AD were ongoing, the Dominantly Inherited Alzheimer Network Trials Unit (DIAN-TU) collaboration was established to test early-stage interventions in patients with dominantly inherited AD (DIAD) caused by mutations in the *APP*, *PSEN1*, or *PSEN2* genes [43]. Because individuals with DIAD have an almost-certain risk of developing AD with a predictable time of onset of symptoms, this specific patient population provides a unique opportunity for evaluating early-stage interventions [44].

Concurrently with the SCarlet RoAD and Marguerite RoAD trials, the DIAN-TU collaboration conducted a separate phase 2/3 trial testing gantenerumab or solanezumab in individuals with DIAD, with the first patients enrolled in late 2012 (Fig. 3C) [45]. This study was initially designed as a 2-year randomized, placebo-controlled, multi-arm trial across asymptomatic (CDR score: 0, cognitively normal) and mild symptomatic (CDR score: 0.5 or 1) disease stages. The primary endpoint was change in PET Aβ deposition for gantenerumab and CSF total (free and bound) Aβ42 concentrations for solanezumab. Gantenerumab was initiated at 225 mg SC every 4 weeks and was later increased to 1200 mg every 4 weeks, based on the SCarlet RoAD futility analysis, subsequent exploratory analyses, and PK/PD modeling as described above [14].

In 2015, the DIAN-TU-001 study was converted into a 4-year clinical efficacy study with a cognitive primary endpoint and biomarker endpoints [14]. The DIAN-TU-001 study did not meet its primary endpoint. Compared to placebo, neither gantenerumab nor solanezumab demonstrated a beneficial effect on cognition in this trial; cognitive change in the clinically normal group was negligible, obfuscating the detection of treatment effects. Gantenerumab significantly reduced Aβ plaques as assessed by Pittsburgh Compound B-PET compared with placebo at 2 (*P* < 0.001) and 4 years (*P* < 0.001). Furthermore, gantenerumab demonstrated significant changes in direction toward normalization on biomarkers of AD pathology and neurodegeneration at year 4, including CSF t-tau (Table 1) [14].

The most common AEs in the 52 participants treated with gantenerumab were injection site reactions (gantenerumab: 47/52 [90%]; placebo: 18/40 [45%]) and nasopharyngitis (gantenerumab: 20/52 [38%]; placebo: 11/40 [28%]). ARIA-E was observed in 10/52 patients (19.2%) in the gantenerumab group and in 1/40 patients (3%) in the placebo group. Of the 11 participants who experienced ARIA-E, 8 were asymptomatic. Symptoms were mild in the remaining 3 participants: 1 experienced headache, 1 experienced dizziness, and 1 experienced a balance disorder with ear pain. All of these symptoms resolved. The mean time for ARIA-E resolution was 85.5 days (SD: 54.3 days), and ARIA-E events were managed by withholding the drug and resuming at similar or lower doses, with most participants reaching the target dose. ARIA-H associated with ARIA-E was seen in 7/52 patients (13%) in the gantenerumab group, and ARIA-H not associated with ARIA-E was seen in 15/52 patients (29%) in this group [14]. Participants from the gantenerumab, solanezumab, and placebo arms of the blinded period of the DIAN-TU-001 study are eligible to receive gantenerumab through the exploratory OLE and will remain blinded to their previous treatment group [14].

## Ongoing phase 3 GRADUATE program

The GRADUATE I and II global, parallel, multicenter, randomized, double-blind, placebo-controlled trials began in 2018 (Fig. 3D) to evaluate the efficacy and safety of SC gantenerumab versus placebo in participants with early AD [34, 46, 47]. Gantenerumab received breakthrough designation from the US FDA in 2021. In these trials, the gantenerumab dose is gradually titrated over 9 months to a target dose of 1020 mg every 4 weeks, administered as 510 mg every 2 weeks, regardless of *APOE ε4* status [34, 47].

The GRADUATE phase 3 clinical trials were designed to incorporate several key learnings from the development program as well as from progress in the field. These considerations include confirmed amyloid positivity (via CSF or PET); optimized exposure to gantenerumab by targeting a single high dose of 1020 mg with a gradual and universal dose-titration regimen, regardless of *APOE ε4* status, to achieve significant Aβ plaque removal while minimizing ARIA-E occurrence; a 24-month study duration (increased to 27 months due to COVID-19) to adequately evaluate clinical outcomes; and study population enrichment to ensure measurable clinical decline [48]. SC administration of gantenerumab improved flexibility and convenience with at-home administration by healthcare professionals [49]. The phase 3 GRADUATE I and II studies are expected to complete in the fourth quarter of 2022 [34, 46]. Eligible participants who complete the GRADUATE studies can enroll in the ongoing POSTGRADUATE OLE study (Table 2) [50, 51].

**Table 2:** Ongoing trials of gantenerumab

Study name, design, and NCT	Study population	Dose	Objective and primary endpoint	Key secondary endpoints	Estimated completion date
**GRADUATE I and II** **Parallel, global, multicenter, double-blind, placebo-controlled, randomized phase 3 studies** (study start date: 2018) (NCT03444870) (NCT03443973)	*N* = 985 (GRADUATE I) *N* = 981 (GRADUATE II) Early (prodromal to mild) AD^**a**^, 50–90 years, evidence of the AD pathological process confirmed by CSF tau/Aβ42 or amyloid PET scan, abnormal memory function, MMSE score ≥ 22, CDR-GS = 0.5 or 1, any *APOE ε4* allele status	Gantenerumab 9-month universal dose-titration regardless of *APOE ε4* allele status to 510 mg SC every 2 weeks (Fig. 3) Placebo	**Objective:** to evaluate the efficacy and safety of subcutaneous gantenerumab vs placebo in patients with early AD (i.e., MCI-AD to mild AD) **Primary endpoint:** change from baseline to week 116 in CDR-SB vs placebo	MMSE, ADAS-Cog 13, Verbal Fluency, Coding, FAQ, ADCS-ADL, safety CSF biomarkers, amyloid and tau PET, MRI, plasma biomarkers	Q4 2022
**Open ROAD** **A long-term, open-label rollover safety and tolerability study of gantenerumab** (study start date: 2020) (NCT04339413)	*N* = 116 Participants who completed Scarlet RoAD OLE (NCT01224106) or Marguerite RoAD OLE (NCT02051608)	Gantenerumab 1200 mg SC Q4W with universal titration (Fig. 3)	**Objective:** to define the long-term safety and tolerability of gantenerumab in patients with AD **Primary endpoint:** AEs, treatment discontinuation, ISR, ARIA-E, ARIA-H, ADAs		May 2025
**GRADUATION** **Multicenter, phase 2, open-label, single-arm, pharmacodynamic study** (study start date: 2020) (NCT04592341)	*N* = 192 Probable mild dementia^**a**^, 50–90 years, AD pathology confirmed by amyloid PET scan	Gantenerumab 255 mg SC Q1W with an option for administration by study partner or non-professional) caregivers (Fig. 3) Placebo	**Objective:** to evaluate the effect of a once-weekly gantenerumab dosing regimen on the change in deposited amyloid **Primary endpoint:** change from baseline in deposited amyloid as measured by brain amyloid PET centiloid levels	Responses to home administration questionnaire (the home administration questionnaire will capture confidence, ease of use, convenience, and overall satisfaction) Safety	November 2023
**DIAN-TU-001 OLE** (study start date: 2020) (NCT01760005)	Patients who complete DIAN-TU-001 (NCT01760005)	Gantenerumab SC uptitrated to 1500 mg Q2W Individuals in the solanezumab arm of the DIAN-TU-001 may switch to gantenerumab	**Objective:** to assess the effects of early and larger magnitude reduction of amyloid plaques on downstream AD processes, the clinical benefits associated with the continued removal of amyloid plaques in DIAD mutation carriers across asymptomatic and symptomatic stages of AD, and the validity of strategies to slow clinical onset of AD and its progression using gantenerumab **Primary endpoint:** change from baseline in: each component of DIAN-MCE; CDR-SB; Functional Assessment Scale; PiB-PET Aβ; CSF total Aβ; CSF t-tau; p-tau181 CSF NfL; precuneus thickness and hippocampal volume Safety		2023
**POSTGRADUATE** **Open-label, multicenter, phase 3 rollover study** (study start date: 2021) (NCT04374253)	Planned *N* = 2032 Participants who completed either GRADUATE I (NCT03444870) or II (NCT03443973) double-blind part or OLE trial	Gantenerumab 9-month universal dose-titration regardless of *APOE ε4* allele status to 510 mg SC every 2 weeks (see Fig. 3)	**Objective:** to define the long-term safety and tolerability of gantenerumab in patients with AD **Primary endpoint:** AEs, SAEs, C-SSRS Score, ARIA-E, ARIA-H, ISRs	CDR, MMSE, ADAS-Cog13, Verbal fluency, Coding, FAQ, ADCS-ADL	December 2024
**PCEx** **Non-interventional, patient- and caregiver-centered qualitative study** (study start date: 2021)	Planned *N* = 100 pairs of patients and their caregivers from the GRADUATE I, GRADUATE II, and POSTGRADUATE studies	Non-interventional	**Objective:** to evaluate the treatment burden associated with gantenerumab for patients and their care partners to optimize the gantenerumab treatment experience in the real world **Primary endpoint:** survey responses of patients’ and caregivers’ experiences with gantenerumab SC administration		October 2022
**SKYLINE** **Phase 3, randomized, double-blind, placebo-controlled secondary prevention trial** (estimated study start date: 2022) (NCT05256134)	Planned *N* = 1200 Cognitively unimpaired, 60–80 years, evidence of cerebral amyloid accumulation, CSF p-Tau_181_/Aβ_42_ ratio > 0.04 or amyloid PET visual read positive; screening includes an optional exploratory BBBM prescreening to predict Aβ positivity	Participant-centric flexible dosing Target gantenerumab dose 1200 mg SC Q1W or Q2W (Fig. 3)	**Objective:** to evaluate the efficacy and safety of gantenerumab in amyloid-positive, cognitively unimpaired patients who are amyloid positive and at risk for AD **Primary endpoint:** PACC-5 Composite endpoint to assess cognition in asymptomatic AD; logical memory from the WMS; FCSRT; coding from the WAIS-IV; MMSE; Category fluency test	CFIa, A-IADL-Q-SV, CDR-SB Safety: MRI, AEs, C-SSRS, ADAs BBBM, vMRI, amyloid and tau PET, CSF biomarkers, pharmacokinetics	October 2028

*Abbreviations*: *Aβ* amyloid-beta, *AD* Alzheimer’s disease, *ADA* anti-drug antibodies, *ADAS-Cog13* Alzheimer’s Disease Assessment Scale–Cognitive Subscale 13, *ADCS-ADL* Alzheimer’s Disease Cooperative Study–Activities of Daily Living instrumental subscale, *AE* adverse event, *A-IADL-Q-SV* Amsterdam Instrumental Activities of Daily Living Questionnaire Short Version, *ARIA-E* amyloid-related imaging abnormalities with edema, *APOE ε4* apolipoprotein E ε4, *ARIA-H* amyloid-related imaging abnormalities with microhemorrhage or superficial siderosis, *BBBM* blood-based biomarker, *CDR-GS* Clinical Dementia Rating Scale–Global Score, *CDR-SB* Clinical Dementia Rating Scale–Sum of Boxes, *CFIa* Cognitive Function Instrument Acute, *CSF* cerebrospinal fluid, *C-SSRS* Columbia-Suicide Severity Rating Scale, *DIAD* dominantly inherited Alzheimer’s disease, *DIAN-MCE* Dominantly Inherited Alzheimer Network–Multivariate Cognitive Endpoint, *DIAN-TU* Dominantly Inherited Alzheimer Network–Trials Unit, *FAQ* Functional Activities Questionnaire, *FCRST* Free and Cued Selective Reminding Test, *ISR* injection site reaction, *MCI* mild cognitive impairment, *mg* milligram, *MMSE* Mini-Mental State Examination, *MRI* magnetic resonance imaging, *NCT* National Clinical Trial number, *NfL* neurofilament light chain, *OLE* open-label extension, *PACC-5* Preclinical Alzheimer’s Cognitive Composite-5 score, *PCEx* patient and caregiver experience, *PET* positron emission tomography, *PiB-PET* Pittsburgh compound-B positron emission tomography, *p-tau* phosphorylated tau, *Q1W* every week, *Q2W* every 2 weeks, *Q4W* every 4 weeks, *SAE* serious adverse event, *SC* subcutaneous, *t-tau* total tau, *vMRI* volumetric magnetic resonance imaging, *WAIS-IV* Wechsler Adult Intelligence Scale 4th edition, *WMS* Wechsler Memory Scale

^a^International Working Group Criteria for Prodromal AD [30]

## Discussion

More than 100 years after the initial description of amyloid plaques, the field of AD research is in a better position to evaluate the amyloid hypothesis, which has been tested using numerous approaches. It is becoming more apparent that removing amyloid can favorably impact AD pathology while promoting beneficial clinical effects [14, 17, 18]. Despite previous disappointments and setbacks in AD research, this is a time of great hope and expectation for clinicians, researchers, patients, and care partners. Several programs have now shown proof of target engagement accompanied by biomarker changes supporting the biological basis for disease modification. Multiple anti-amyloid monoclonal antibodies that fully remove Aβ plaques have demonstrated cognitive and clinical benefits in early AD. The availability of SC administration allows greater convenience in the doctor’s office or at home.

The extent to which biomarkers will ultimately predict clinical outcomes of drug treatment remains to be seen as new data emerge. From a US FDA perspective—though not from a global regulator standpoint—amyloid PET is recognized as a surrogate that is reasonably likely to predict a clinical benefit to patients, pending a required post-approval trial to verify that the drug provides the expected clinical benefit [6, 52]. The GRADUATE studies will evaluate clinical benefit/risk in a large population of patients with early AD and will provide a rich data source to investigate the relationships between previously studied and newer biomarkers and clinical outcomes. In particular, the inclusion of tau PET in the GRADUATE studies may move this biomarker closer to broader acceptance by health authorities as a surrogate biomarker.

The disease stage that should be targeted for optimal treatment effect using amyloid-directed therapies is still uncertain, but likely will include removing amyloid before cognitive symptoms appear. Ongoing primary and secondary prevention trials will help address the optimal stage of the disease to remove or prevent Aβ plaques and the impact of this removal in disease progression. The future of the field involves identifying the key disease stage for treatment and target engagement using methods to increase central nervous system exposure, as well as considering other therapies and modalities (e.g., anti-tau therapies) and even combination therapies, including a brain shuttle that combines gantenerumab with a transferrin receptor 1 binding “Brain Shuttle” module, enabling active receptor-mediated transport across the blood-brain barrier.

## Conclusions

Gantenerumab trials have greatly informed the past, present, and future of AD therapeutic research. The early trials did not meet their primary objectives; however, they contributed valuable knowledge that informed subsequent trials on dosing, titration, route of administration, patient population, and the effect of amyloid-lowering on downstream biomarkers of disease pathology. As demonstrated over the past 22 years, the road to understanding and treating AD may be circuitous and difficult. Each clinical trial—regardless of the outcome—ultimately leads to greater knowledge and greater power to gain control over a terrible disease.

## Data Availability

The data that support the findings of this study are available from the corresponding author, RJB, upon reasonable request.

## Abbreviations

^18^F-FDG-PET: ^18^F-fluorodexyglucose positron emission tomography
Aβ: Amyloid beta
Aβ(1–40): Amyloid beta protein fragment 1-40
Aβ(1–42): Amyloid beta protein fragment 1-42
AD: Alzheimer’s disease
ADA: Anti-drug antibody
ADAS-Cog13: Alzheimer’s Disease Assessment Scale-Cognitive Subscale 13
ADCS-ADL: Alzheimer’s Disease Cooperative Study-Activities of Daily Living
AE: Adverse event
A-IADL-Q-SV: Amsterdam Instrumental Activities of Daily Living Questionnaire Short Version
AICD: Amyloid precursor protein intracellular domain
*APOE ε4*: Apolipoprotein E ε4
*APP*: Amyloid precursor protein
ARIA: Amyloid-related imaging abnormalities
ARIA-E: Amyloid-related imaging abnormalities with edema
ARIA-H: Amyloid-related imaging abnormalities with microhemorrhage or superficial siderosis
BACE: Beta-secretase
BBBM: Blood-based biomarker
CDR: Clinical Dementia Rating
CDR-GS: Clinical Dementia Rating Scale–Global Score
CDR-SB: Clinical Dementia Rating Sum of Boxes
CFIa: Cognitive Function Instrument Acute
CPR: Cognitive progression ratio
CSF: Cerebrospinal fluid
C-SSRS: Columbia-Suicide Severity Rating Scale
DIAD: Dominantly inherited Alzheimer’s disease
DIAN-MCE: Dominantly Inherited Alzheimer Network–Multivariate Cognitive Endpoint
DIAN-TU: Knight Family Dominantly Inherited Alzheimer Network Trials Unit
DOF: Data on file
FAQ: Functional Activities Questionnaire
FCRST: Free and Cued Selective Reminding Test
FDA: Food and Drug Administration
HuCAL: Human Combinatorial Antibody Library
ISR: Injection site reaction
IV: Intravenous
LS: Least squares
MAD: Multiple ascending dose
MCI: Mild cognitive impairment
MMRM: Mixed models for repeated measures
MMSE: Mini-Mental State Examination
MOA: Mechanism of action
MR: Marguerite RoAD
MRI: Magnetic resonance imaging
MR-NP: Marguerite RoAD non-pretreated cohort
MR-P: Marguerite RoAD pretreated cohort
NCT: National Clinical Trial number
NfL: Neurofilament light chain
OLE: Open-label extension
PACC-5: Preclinical Alzheimer’s Cognitive Composite-5 score
PCEx: Patient and caregiver experience
PD: Pharmacodynamic
PET: Positron emission tomography
PiB-PET: Pittsburgh compound-B positron emission tomography
PK: Pharmacokinetic
*PSEN1*: Presenilin-1
*PSEN2*: Presenilin-2
p-tau: Phosphorylated tau
p-tau181: Phosphorylated tau181
Q1W: Every week
Q2W: Every 2 weeks
Q4W: Every 4 weeks
SAE: Serious adverse event
sAPPβ: Soluble amyloid precursor protein-beta
SC: Subcutaneous
SD: Standard deviation
SE: Standard error
SR: SCarlet RoAD
T: Tau
t-tau: Total tau
vMRI: Volumetric magnetic resonance imaging
WAIS-IV: Wechsler Adult Intelligence Scale 4th edition
WMS: Wechsler Memory Scale
